# Instrumentation for quantitative analysis of volatile compounds emission at elevated temperatures. Part 2: Analysis of carbon fibre reinforced epoxy composite

**DOI:** 10.1038/s41598-020-65473-4

**Published:** 2020-05-26

**Authors:** Célia Lourenço, Daniel Francis, Dawn P. Fowler, Stephen E. Staines, Jane Hodgkinson, Christopher Walton, Sarah Bergin, Ralph P. Tatam

**Affiliations:** 1 Centre for Engineering Photonics, Cranfield University, Cranfield, Bedfordshire MK43 0AL UK; 2 Environmental Analytical Facility, Cranfield University, Cranfield, Bedfordshire MK43 0AL UK; 3 Centre for Environmental and Agricultural Informatics, Cranfield University, Cranfield, Bedfordshire MK43 0AL UK; 4 Present Address: HSE Science and Research Centre, Harpur Hill, Buxton, Derbyshire, SK17 9J UK

**Keywords:** Composites, Techniques and instrumentation

## Abstract

We have investigated the release of gases and volatile organic compounds (VOCs) from a carbon fibre reinforced epoxy composite matrix used in aircraft structural components. Analysis was performed at several temperatures both up to and above the recommended operating temperature (121 °C) for the material, to a maximum of 250 °C. Gas chromatography-mass spectrometry (GC-MS) combined with thermal desorption (TD-GC-MS) was used to identify and quantify VOCs, and in parallel real-time gas detection with commercial off-the-shelf (COTS) gas sensors. Under hydrocarbon free air, CO, SO_2_, NO, NO_2_ and VOCs (mainly aldehydes, ketones and a carboxylic acid) were detected as the gaseous products released during the thermal exposure of the material up to 250 °C, accompanied by increased relative humidity (4%). At temperatures up to 150 °C, gas and volatile emission was limited.

## Introduction

Composite materials are often required to perform in demanding environments and subject to complex environmental conditions such as in the aircraft structure. The primary advantages of composite materials are their high strength, relatively low weight and corrosion resistance^1^. A fibre composite material consists of an array of fibres often in a polymeric (e.g. polyester, phenolic, epoxy) thermosetting matrix. Epoxy-based composite materials are widely used as, for example, laminates for aerospace, ballistic, engineering components, typically containing epoxide groups (C–O–C ring structure) on the backbone structure of the resin. The thermal stability of carbon fibre reinforced epoxy-based polymeric matrix is affected by the structure of the particular epoxy resin under study (i.e. the epoxy monomer); the chemical nature of the curing agent/hardener and the crosslink density; the curing schedule (with peak temperatures typically varying from 160 °C to 180 °C); the type of fibre and fibre content used within the matrix; the environmental oxygen concentration and moisture content^2–6^. The matrix resin may also include performance enhancing agents such as blends of thermoplastic particles which are often added to provide greater damage tolerance and interlaminar toughness^7^; flame retardants which directly impact on its glass transition temperature (Tg) that typically varies from 120 °C to 190 °C^1,8^; viscosity modifiers since flame retardants application is usually accompanied by increased polymer matrix viscosity^8^.

Studies investigating the thermal decomposition of carbon fibre epoxy composites have been reported in the literature comprising the use of pyrolysis and thermogravimetric (TG)^8,9^ measurements coupled with gas analysers, such as Fourier Transformed Infrared spectroscopy (FTIR)^3,10,11^ or Gas Chromatography-Mass Spectrometry (GC-MS)^12,13^; cone calorimetry used to evaluate the mass loss of the material throughout the test and where CO and CO_2_ concentrations are measured in the exhaust duct^14,15^; and the modelling of the thermodynamic properties and/or kinetic behaviour^16,17^ has been considered.

In the aeronautics industry, one of the concerns is the thermo-oxidative stability of composite materials due to high temperature exposure. Tight safety regulations are set in place for commercial aircraft, including air quality^18^. The aircraft cabin air quality has been paid attention in recent years due to potential health implications, as passengers and crew are confined to the aircraft cabin with low humidity and reduced air pressure, and potential for exposure to contaminants such as volatile organic compounds (VOCs). In-flight measurements of VOCs have been reported although there has been no conclusive evidence for target pollutants occurring in the cabin air at levels exceeding available health and safety standards and guidelines^19–23^.

With the introduction of increasing levels of carbon fibre reinforced composite (CFRC) within aircraft, there is potential for this to add to the gases and volatiles present within cabins. In this study we investigated the gaseous emissions arising from the thermal exposure both up to and above the recommended operating temperature (121 °C) for the material to a maximum of 250 °C of a carbon fibre reinforced epoxy composite material T700GC/M21 – which is widely used in the aeronautics industry – through the use of commercial off-the-shelf (COTS) gas sensors (implemented and fully described in Part 1) and sorbent tubes further analysed by Gas Chromatography-Mass Spectrometry coupled to Thermal Desorption (TD-GC-MS). The quantitative analysis of emission from materials within a closed chamber at controlled flow rates is considered to be a first step that would enable the estimation of potential emissions from these materials in service, in standard operation and at elevated temperatures.

## Results and discussion

The composite material T700GC/M21 has been developed to operate in environments up to 121 °C and is reported to have good hot/wet (high temperature/humidity) properties up to 150 °C^24^. There is evidence that the material T700GC/M21 thermally decomposes in three different steps under oxidative atmosphere, i.e. (1) decomposition of M21 resin (up to 400 °C), (2) thermo-oxidation of the carbonaceous residue (400 °C–650 °C), and (3) thermo-oxidation of the T700 fibres (650 °C–1000 °C)^3^. In addition, the decomposition of the resin was found not to be influenced by the presence of oxygen up to 400 °C, namely, the comparison of the thermogravimetric curves under nitrogen or air revealed identical results up to 400 °C. Weight loss was reported above 100 °C^3,25^. Thermogravimetric Fourier transformed infrared spectroscopic (TG-FTIR) measurements identified H_2_O, PA6, phenol, CH_4_, COS and CO as the main species released^3^. Supporting the earlier evidence, previous work reported a melting peak at 190 °C attributed to the melting of a thermoplastic element of the matrix^16^.

### Mass loss measurement

**Figure 1:**
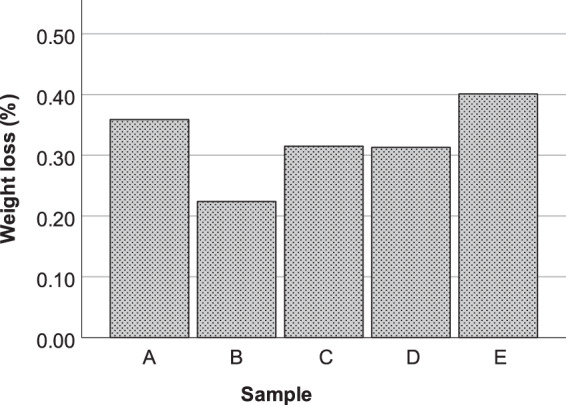
Individual weight loss (%) for composite material T700GC/M21 obtained at 250 °C. Mean weight loss of 0.32%.

In the present study, a mean weight loss of 0.32% was observed at the end of the test (Fig. 1) following heating in stages to 250 °C. Weight losses are consistent with previously reported data for the same material (under air), where pronounced weight loss (18%) was reported between 400 °C and 500 °C, and at lower temperatures a negligible weight loss was observed^3^.

### Real-time sensor measurements

As the temperature rises the organic matrix undergoes degradation in the sequence of endothermic reactions usually by either random chain scission, end-chain scission, and chain stripping, yielding low molecular weight gaseous products^26^. The main gaseous products (H_2_O, degradation products of PA6, phenol, CH_4_, COS and CO) released during the epoxy composite pyrolysis are known and have been previously reported^3^. Under an oxygen atmosphere, it was previously reported that the thermoplastic blend (polyether sulfone (PES) and polyamide (PA6)) decompose during the two first steps of degradation (up to 650 °C)^3^.

**Figure 2:**
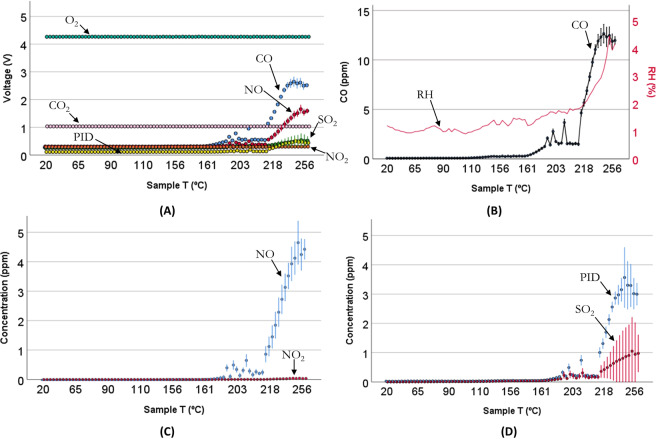
Response of the sensors upon exposure to the gas sample **(A)** Output voltages expressed in Volts (V) **(B)** Dual axis representation of mean CO concentration (ppm) and mean relative humidity [RH (%)] detected over the sample temperature **(C)** Respective mean gas concentration expressed in parts-per-million (ppm) for NO and NO_2_ gas emissions **(D)** PID (volatiles) and SO_2_ plots respectively. Error bars represent ± 2 s.e.m. (standard error of mean), n = 5 samples, batch 1.

In our experimental conditions, CO, SO_2_, NO, NO_2_ and VOCs were detected as the gaseous products released during the thermal degradation process (Fig. 2). Higher temperatures promote the formation of CO_2_, which is particularly dependent on oxygen availability to the combustion^27^. In this study no significant response was observed for the CO_2_ sensor.

Under hydrocarbon free air, no consumption of oxygen (Fig. 2) was detected within the temperature range investigated in this paper, in agreement with a previous study where a pure epoxy uncured resin was studied^28^.

The response of the sensor array (Fig. 2) upon exposure to the gas sample has shown the CO sensor first response (baseline voltage ± 0.005 V) at a sample temperature of 116 °C. This was followed by the SO_2_ gas sensor at 147 °C, the PID sensor at 173 °C, the NO sensor at 185 °C and lastly the NO_2_ sensor at 202 °C.

**Figure 3:**
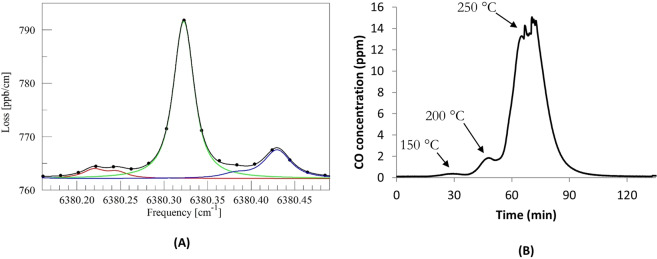
CO measurement using Picarro cavity ringdown spectrometer **(A)** Near infrared spectrum recorded using tunable laser. Green line indicates CO optical absorption spectrum and the black line is the best fitted model of the spectrum at 250 °C. Red line indicates CO_2_ optical absorption spectrum, and blue line indicates H_2_O optical absorption spectrum **(B)** concentration plot expressed in parts-per-million (ppm) detected for CO (21 °C– 250 °C).

As the sample temperature rises inside the tube furnace, the CO levels increase substantially from 200 °C onwards, reaching 12 ppm ± 0.2 ppm CO at 250 °C, accompanied by increased relative humidity (4%) to the furnace outlet (Fig. 2). Emission of CO is consistent with typical thermal-oxidative degradation schemes of epoxy resins that have been proposed and reported^29,30^. Carbon monoxide emissions can result from the cleavage of epoxide groups [epoxide ring → –(HC=CH)– + H_2_O + CO], i.e. the epoxide ring opens up followed up by the loss of one H_2_O and one CO molecule. The level of excess water vapour observed is too great to be accounted for by degradation of the epoxy matrix via this mechanism. However, the influence of moisture uptake at room temperature before analysis is unknown and it is possible that there was vaporisation of moisture already present in the polymer matrix. The increased level of water vapour measured at temperatures above 200 °C is considered too high to be surely associated with degradation pathways to CO and H_2_O. The concentration results (12 ppm ± 0.2 ppm CO at 250 °C) are in good agreement with the CO measurement using the Picarro spectrometer (Fig. 3), the latter peaking at 14 ± 1 ppm over the peak period from 65 min to 73 min. The results therefore support and validate the findings related to CO production and confirm that any cross-response of the electrical CO sensor to other gases or volatiles emitted from the samples was negligible in comparison. Relative differences in CO emissions levels appear to be associated to the material thickness^14^ and the use of flame retardants^28^.

Although human endurance limits listed in Table 1 (permissible exposure limits, PELs) are applicable in standard atmospheric conditions – and not the reduced pressure and humidity that represent the typical cabin air environment – this is presented here for context due to the lack of specific guidance for aircraft limits for species other than CO and CO_2_. These findings indicated that carbon monoxide (CO) concentration released from the composite material is below its permissible 8-hour exposure limit (PEL) which is known at 25 ppmv^31^, whereas the CO limit for aircraft crew and passengers is set at 50 ppmv (Table 1). Although, modelling is needed to link the emission rate in such tests with a possible concentration in the cabin, since area and dilution effects need to be considered.

**Table 1:** Comparative list of the approved permissible exposure limits (PELs) by Occupational Safety and Health Administration (OSHA), and the code of federal regulations (CFR) FAA (Federal Aviation Authority) limits for aircraft.

Substance	Molecular formula	OSHA PEL (ppmv)^a^	FAA limits for aircraft (ppmv)^b^
Acetaldehyde	C_2_H_4_O	25	n/a
Acetic acid	C_2_H_4_O_2_	10	n/a
Acetone	C_3_H_6_O	500	n/a
Acrolein	C_3_H_4_O	0.1	n/a
2-Butanone	C_4_H_8_O	200	n/a
Carbon monoxide	CO	25	50
Carbon dioxide	CO_2_	5000	5000
Nitric oxide	NO	25	n/a
Nitrogen dioxide	NO_2_	1	n/a
Sulfur dioxide	SO_2_	2	n/a

“n/a” stands for non-applicable. Concentrations are expressed in parts-per-million by volume (ppmv).

^a^OSHA permissible exposure limits (PELs). Time weighted average (TWA)^31^.

^b^Code of federal regulations (CFR) FAA (Federal Aviation Authority) limits for aircraft^18^.

Nitric oxide (NO) gas detection has been previously reported as one of the main gaseous emissions yielded by the thermal degradation of an epoxy resin/carbon fibre composite^32^. Nitric oxide (NO) levels (Fig. 3) increased up to 4 ppm ± 0.2 ppm NO (250 °C) in the experimental study reported here.

Although we have not observed a significant drop in O_2_ concentration throughout the tests, it is likely that the NO_2_ minor signal (<0.1 ppm at 200 °C onwards) is a reaction product via the oxidation of nitric oxide by oxygen in air (2NO + O_2_ → 2NO_2_) (Fig. 3). The fact that oxygen levels remained approximately constant confirms that oxidative processes took place in an oxygen concentration representative of standard conditions.

The detection of SO_2_ gas emissions (2 ppm ± 0.3 ppm SO_2_ at 250 °C) is attributed either to the thermal degradation of the curing agent 4,4’-diaminodiphenyl sulfone (commonly known as DDS) and/or that of polyether sulfone (PES) these being the main sulfur-containing elements of the composite^33,34^.

The emission of VOCs with ionisation potentials (IE) < 10.6 eV were detected with the PID sensor at 3 ppm ± 0.4 ppm at 250 °C (Fig. 3). The PID sensor has known cross-response to a wide range of volatiles and it was calibrated with isobutylene, therefore its output is referenced to this gas^35^. Thus, suitable identification and quantification is required using other techniques, such as the use of sorbent tubes further desorbed with GC-MS.

A sixth sample obtained from an independent batch (batch 2) was tested to account for batch-to-batch variation (Table 2) on gaseous emissions. No significant response was observed for the CO_2_ sensor. Minor variance was observed between the samples (n = 6) and over the temperature, including the sixth sample acquired from a different batch number. This supports the premise that the gaseous emissions are real and not an artefact of a faulty batch.

**Table 2:** Sample (n = 6) variance over the sample temperature (T °C) and investigation of batch-to-batch variation on gaseous emissions.

T (°C)	Variance					
	CO	NO	SO_2_	PID	NO_2_	O_2_
70	0.001	0.000	0.000	0.000	0.000	0.007
150	0.001	0.000	0.000	0.000	0.000	0.007
200	0.209	0.022	0.011	0.006	0.000	0.006
250	1.170	0.490	1.209	0.475	0.001	0.008

### Gas-phase analysis using TD-GC-MS

**Figure 4:**
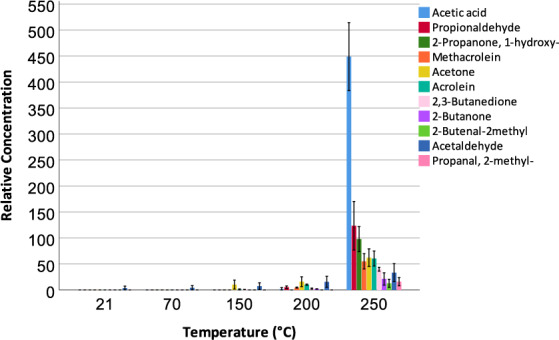
Volatile profile released as a function of temperature and determined using TD-GC-MS. Error bars represent ± 2 s.e.m. (standard error of mean) over all five samples, n = 5, batch 1.

In this study, volatile organic compounds (VOCs) released throughout the thermal exposure of the material were characterised using gas chromatography-mass spectrometry combined with thermal desorption (TD-GC-MS), wherein the gas was sampled into sorbent tubes at specific time points, i.e. 21 °C, 70 °C, 150 °C, 200 °C and 250 °C. An untargeted analysis (semi-quantitative) was conducted and VOCs relative concentrations were determined (Fig. 4). Emissions rates expressed in mg m^−2^ min^−1^ were determined for the VOCs identified in this study (Table 3).

**Table 3:** Emission rates (mean, n = 5, batch 1), expressed in mg m^−2^ min^−1^ (i.e. normalised to 1 m^2^ area of composite material), at different temperatures for volatile organic compounds (VOCs) and other gases identified in this study; chemical abstract service (CAS) registry number of compounds and respective molecular formulae are provided.

Compound	Molecular formula	CAS	Relative Emission rate (mg m^−2^ min^−1^)				
			21 °C	70 °C	150 °C	200 °C	250 °C
Acetic acid	C_2_H_4_O_2_	64-19-7	0	0	0	0	340
Propionaldehyde	C_3_H_6_O	123-38-6	0	0	0	3.2	110
2-Propanone, 1-hydroxy-	C_3_H_6_O_2_	116-09-6	0	0	0	0	70
Methacrolein	C_4_H_6_O	78-85-3	0	0	0	4.0	50
Acetone	C_3_H_6_O	67-64-1	0	0	7.2	11	50
Acrolein	C_3_H_4_O	107-02-8	0	0	0	8.0	54
2,3-Butanedione	C_4_H_6_O_2_	431-03-8	0	0	0.8	1.6	30
2-Butanone	C_4_H_8_O	78-93-3	0	0	0	1.6	18
2-Butenal-2-methyl	C_5_H_8_O	1115-11-3	0	0	0	0	10
Acetaldehyde	C_2_H_4_O	75-07-0	0.8	0.8	3.2	14	34
Propanal, 2-methyl	C_4_H_8_O	78-84-2	0	0	0	0	12
			**Absolute Emission rate (mg m**^**−2**^ **min**^**−1**^**)**				
Carbon monoxide	CO	630-08-0	0	0	200	1100	9900
Nitric oxide	NO	10102-43-9	0	0	0	110	2700
Sulfur dioxide	SO_2_	7446-09-5	0	0	24	88	600
VOCs	PID	n/a	0	0	32	160	2400
Nitrogen dioxide	NO_2_	10102-44-0	0	0	0	0	24

In this study, several VOCs were detected as the major VOCs released during the thermal degradation process, including acetic acid; propanal (commonly known as propionaldehyde); 2-propanone, 1-hydroxy-; methacrolein; acetone; 2-propenal (commonly known as acrolein); 2,3-butanedione; 2-butanone; 2-butenal, 2-methyl; acetaldehyde; propanal, 2-methyl. Earliest detection of VOCs was triggered by the PID sensor at 173 °C consistent with the TD tube analysis at 200 °C where initial detection of VOCs was observed. Apart from acetaldehyde, the statistically significant increase (p < 0.05) in VOCs was observed between the time points 200 °C–250 °C. The released VOCs identified in this study are consistent with previous work on thermal-oxidative degradation schemes of epoxy resins, where mostly propionaldehyde, carbon monoxide, acrolein and/or ethylene, acetaldehyde, formaldehyde, acetone or propylene were proposed to be present in the volatile mixture^29^. The results obtained from the sixth sample tested (independent batch 2) is in agreement with the previous findings.

Under environments operated up to 121 °C, the recommended maximum operating temperature of the material, the composite material does not contribute to the VOCs in the cabin. In a fire situation, such as in-flight and post-crash fires, VOCs are expected to build-up as soon as the temperature of the material reaches 173 °C. The permissible 8-hour exposure limits (PELs) currently regulated by Occupational Safety and Health Administration (OSHA) are listed in Table 1. On-board air quality governed by Federal Aviation Authority (FAA) confines only CO and CO_2_ gas emissions to 50 ppmv and 5000 ppmv respectively.

### Summary of gas emission

Table 3 summarises the emission rates of all the gases and VOCs mentioned above in units of mg m^−2^ min^−1^. In order to permit modelling of the cabin environment, these figures should be considered indicative of the emission from a sample of the dimensions given above (50 × 25 mm, 4 mm thickness). In order to assess any effect on cabin air quality, modelling would be required that links the emission rates with the flow of circulating air and the area of material affected by heat. Nevertheless, these results confirm that up to the maximum recommended operating temperature of the material analysed, emission of gases or volatiles was negligible, with only a small amount of acetaldehyde detected and minor CO emission. The emissions rates for gases and VOCs were estimated considering an inlet flow of 1000 cm^3^ min^−1^. The authors estimate that errors in the flow rate used to calculate the release rate of both gases and VOCs is minimal. A possible source of error is that the air passing through the furnace was heated by up to 1 °C during the experiments and would therefore have slightly expanded at the outlet. The accuracy of the mass flow controller itself is specified to be 0.75%.

## Conclusion

This study aimed to investigate the influence of thermal exposure of carbon fibre reinforced epoxy composite on the production of gaseous emissions and its potential for impact on the on-board air quality. A parallel approach was used, employing real-time gas detection accomplished with commercial gas sensors and the use of sorbent tubes further analysed by TD-GC-MS. The full details of this method have been provided in Part 1 of this paper.

A mean weight loss of 0.32% was observed, which is in line with previously reported weight losses for the same material. When compared with emission rate data, the magnitude of the weight loss is too great to be accounted for by degradation of the epoxy, so may be attributed to loss of adsorbed water, a conclusion that is also supported by the levels of excess relative humidity observed. Under hydrocarbon free air, CO, SO_2_, NO, NO_2_ and VOCs were detected as the gaseous products released during the thermal exposure of the material up to 250 °C. The identified VOCs included acetic acid; propanal (commonly known as propionaldehyde); 2-propanone, 1-hydroxy-; methacrolein; acetone; 2-propenal (commonly known as acrolein); 2,3-butanedione; 2-butanone; 2-butenal, 2-methyl; acetaldehyde and propanal, 2-methyl. Thermal desorption is a pre-concentration stage for gas chromatography, thus the concentration levels determined do not correlate to the actual levels in the cabin, flow rates and areas also differ from the actual aircraft cabin environment. Therefore, modelling is needed to link the emission rate in such tests with a possible concentration in the aircraft cabin and we have also reported our results as emission rates in units of mg m^−2^ min^−1^.

The understanding of the oxygen role during the degradation process is crucial to predict service life of the material and maximum safe operating temperature under standard operational conditions. Chemical identification and quantification of gaseous emissions, in particular VOCs, helps implementing proper safety protocols. So far, most reported studies have focused their attention on the study of gaseous emissions from epoxy resins under inert atmosphere, and quantitative data is limited. To the best of our knowledge, this is the first time simultaneous analysis of gases is accomplished (not only low molecular weight gaseous products but also volatile organic compounds (VOCs)), under a standard air atmosphere and throughout the heating process. Our results also act as a confirmation of the applicability of COTS sensors to detect gases that may be emitted from aerospace composite that is experiencing excessive levels of heat, above its maximum operating temperature.

## Methods

The instrumentation fully described in Part 1 of this paper, allowed the characterisation of composite materials inside an air-tight horizontal tube furnace (Carbolite Gero EHA 12/300B/200) operated up to 250 °C. The test system was designed to provide a controlled flow (1000 cm^3^ min^−1^) of hydrocarbon free air (BOC products 200 bar cylinder) through the furnace. The furnace temperature ramp was set at a rate of 5 °C min^−1^ with 10 min dwell points at 70 °C, 150 °C, 200 °C and 250 °C, and after completing the temperature programme the system was shut down. The temperature inside the work tube (“*furnace temperature*”) was recorded every 60 seconds using Eurotherm iTools software. Independent temperature measurement of the sample (“*sample temperature*”) was monitored in a separate experiment, using a K-type thermocouple (temperature range -60 °C to +350 °C, RS Components) inserted into the sample and a temperature data logger (Pico Technology USB TC-08) recorded the average temperature every 60 seconds.

The system included pressure (P), temperature (T) and relative humidity (RH) control at downstream locations within the system and this was used to monitor the gases downstream of the tube furnace and before passing through the sensors.

Real-time detection of released gases was accomplished using commercial off-the-shelf (COTS) gas sensors supplied by Alphasense. Data acquisition was automatically performed using LabVIEW 2014 software. Furthermore, stainless-steel thermal desorption (TD) tubes (Markes International Ltd) were used simultaneously and later analysed by gas chromatography-mass spectrometry combined with thermal desorption (TD-GC-MS) for further qualitative and semi-quantitative analysis. Experimental results for a CO sensor were validated using a Picarro cavity ringdown spectrometer.

Alumina combustion boats (119 × 30 × 19 mm, Avon Green Scientific) were baked off at 700 °C for 24 hours prior to use and cooled to ambient temperature inside a desiccator. The sample weight and weight of the empty boat was recorded prior to the study (balance Ohaus GA200D ± 0.0001 g accuracy), followed by the weight record of the overall system (boat** +** sample) at the end of the experiment. The weight loss (%) released throughout the heating process up to 250 °C was determined at the end of the test. Loading and unloading of the furnace was strictly performed at room temperature. The sample was sitting at an angle of 30° over the combustion boat. The set “boat + sample” was placed at the centre of the isothermal zone, and the end seals firmly closed. A high temperature cleaning procedure was employed after each test and a blank test was performed prior to each experiment to confirm that the apparatus and hydrocarbon free air were uncontaminated.

### Materials

Six samples (50 × 25 mm, 4 mm thickness) were made of a standard carbon fibre reinforced composite material Hexcel Hexply M21/35%/268/T700GC (resin/resin content by weight (%)/fibre weight (gsm)/fibre type), a high strength carbon based fibre with a third generation toughened epoxy resin matrix. The samples were taken from the sample batch and cut from a larger sheet of material by three axis CNC (computer numerical controlled) milling. Care was taken to ensure negligible contamination of the samples during cutting or subsequent handling. The M21 epoxy resin formulation is constituted of three types of epoxy resin diGlycidyl ether bisphenol F (known as DGEBF), triglycidylether *meta*-aminophenol (known as T-GMAP), and *para*-glycidyl amine); one hardener (4,4’-diaminodiphenyl sulfone, commonly known as DDS); and thermoplastic blends (polyether sulfone (PES) and polyamide (PA6/PA12)^4,10^. The composite was fabricated from a standard carbon fibre prepreg, which may have employed unspecified sizing agents to promote adhesion between the carbon fibres and the epoxy matrix, and was processed via standard conditions including potential release agents used in the mould. The first five samples (batch 1) were obtained from the same manufactured batch of material and the sixth sample (batch 2) was obtained from an independent batch in order to check for batch-to-batch variation.

### COTS gas sensors

Commercial gas sensors (Alphasense) included a photoionisation detection (PID) gas sensor (PID-AH2) (VOCs); a non-dispersive infrared (NDIR) sensor for carbon dioxide (IRC-A1 CO_2_); electrochemical sensors including nitric oxide (NO-A4), nitrogen dioxide (NO_2_-A43F), sulfur dioxide (SO_2_-A4), carbon monoxide (CO-A4); and oxygen (O_2_-A2) sensor.

All the sensors were pre-calibrated at Alphasense. The sensors sensitivity was determined and listed in Table 4.

**Table 4:** Sensor specifications including limit of detection (LOD) and determined sensor sensitivities expressed in V/ppb.

Sensor	Type of sensor	LOD (ppb)	Sensitivity (V/ppb)
PID	photoionisation	1	1.10 × 10^−4^
NO_2_	electrochemical	15	2.02 × 10^−4^
SO_2_	electrochemical	15	2.69 × 10^−4^
NO	electrochemical	80	2.96 × 10^−4^
CO	electrochemical	20	1.89 × 10^−4^
CO_2_	non-dispersive infrared	0% vol	n/a
O_2_	Electrochemical	15%	n/a

The absorbance as a function of gas concentration is non-linear and the linearised gas concentration (in % volume) was set within the LabVIEW programme and the sensor calibrated at 0-20% Vol CO_2_. The O_2_ sensor was calibrated in ambient air, considering reliably 20.9% oxygen in air. Gas concentrations were determined within the LabVIEW programme accordingly.

The gas sensors’ working principle and implementation was fully described in Part 1 of this paper.

### TD-GC-MS

Volatile organic compounds (VOCs) were trapped into pre-conditioned stainless-steel sorbent tubes for 5 min at a controlled flow of 100 cm^3^ min^−1^. Samples were taken at different time points, corresponding to sample temperatures of 21 °C, 70 °C, 150 °C, 200 °C and 250 °C and further analysed by GC-MS combined with thermal desorption (TD-GC-MS). The TD-GC-MS principle is well documented elsewhere^36^. The tubes were pre-conditioned with dual packing comprising 40% Tenax and 60% Carbotrap (Markes International Ltd). Prior to analysis, the tubes were spiked with 0.5 µl of internal standard, d8-toluene in methanol (100 ng μl^−1^), and then flushed with helium for 3 min for further semi-quantitative analysis.

Chromatographic analyses were performed using a GC Agilent 7890 A TOF-MS system (Bench ToF –dx (DS)) equipped with a Markes ULTRA TD autosampler, and Markes UNITY thermal desorber. The volatiles were separated using a Restek column Rxi-624 Sil MS (60 m × 0.25 mm, film thickness 1.4 µm) working in a constant flow mode with a temperature ramp. The tubes underwent a pre-purge of 1.0 minute, followed by desorption at 300 °C for 8.0 min. The initial trap temperature was set at -10 °C and the actual trap desorption occurred at 300 °C for 3.0 min. The column temperature program involved an initial dwell at 35 °C for 1.0 minute, followed by an increase from 35 °C to 75 °C at a rate of 2 °C min^−1^, followed by a ramp from 75 °C to 140 °C at a rate of 5 °C min^−1^, from 140 °C to 300 °C at a rate of 10 °C min^−1^, and a constant temperature of 300 °C for 12 min.

The mass spectrometer was operated in an associated mass-to-charge ratio (*m/z*) range set from 34 to 350. The ion source and transfer line temperature were kept at 200 °C and 150 °C, respectively. GC-MS data analysis was performed using AMDIS (Automated Mass Spectral Deconvolution and Identification System) software and followed by reliable identification of compounds using the NIST (National Institute of Standards and Technology) library. Statistical analysis was performed using the software IBM SPSS Statistics 25.0.

### Picarro cavity ringdown spectrometer

Electrochemical sensors can have the potential to cross-respond to other gases. The CO sensor used in this work included a filter to reduce such cross-response, but nevertheless it was decided to use a Picarro cavity ringdown spectrometer (Picarro Inc., CA, USA, model G2401 for CO_2_** +** CO** +** CH_4_** +** H_2_O) to validate the electrically measured carbon monoxide concentrations.

The cavity ringdown spectroscopy (CRDS) technique exhibits enhanced sensitivity in the parts-per-million to parts-per-trillion (ppb-ppt) range to detect small molecules such as carbon monoxide (CO) via measurement of the near-infrared absorption spectrum^37^. Light from a tunable semiconductor diode laser is directed into an optical cavity (analyte gas) consisting of three highly reflective mirrors which provides an effective optical pathlength of several kilometres. A photodetector is situated behind the last mirror, measuring the light intensity and specifically its energy decay is measured as a function of time, known as “*ringdown*”. The *ringdown* time acts as a sensitive measure of optical absorption and profiles are transformed into an absorption spectrum, an example of which is shown in Fig. 3 (A). Gas concentration is determined by a multi-parameter fit to an absorption line shape^38^. The technique is known to have negligible cross-sensitivity to other gases as a result of the high spectral resolution employed.

Ambient air stabilisation was allowed prior to analysis. The sensor system exhaust duct was connected to the sampling inlet of the Picarro spectrometer, and exhaust gases were then safely vented into the fume cupboard. Calibrated gas concentrations expressed in parts-per-million (ppm) were drawn for CO measurement.

## Data Availability

The datasets generated during and/or analysed during the current study are available in the Cranfield Online Research Data (CORD) repository, [10.17862/cranfield.rd.9805427].
